# Mediastinal perigraft seroma after thoracic aortic surgery

**DOI:** 10.1093/icvts/ivad148

**Published:** 2023-09-04

**Authors:** Ryo Suzuki, Masafumi Akita, Suguru Miyazaki, Ryo Shimano

**Affiliations:** Department of Cardiovascular Surgery, Shinmatsudo Central General Hospital, Matsudo, Japan; Department of Cardiovascular Surgery, Shinmatsudo Central General Hospital, Matsudo, Japan; Department of Cardiovascular Surgery, Shinmatsudo Central General Hospital, Matsudo, Japan; Department of Cardiovascular Surgery, Shinmatsudo Central General Hospital, Matsudo, Japan

**Keywords:** Mediastinal perigraft seroma, Thoracic aortic surgery, Perigraft fluid collection, Seroma after vascular surgery

## Abstract

**OBJECTIVES:**

We investigated pertinent factors associated with mediastinal perigraft seroma (PGS) after thoracic aortic surgery. In addition, we provided a clinical review of this entity, as reports reviewing abundant mediastinal PGS cases are rare.

**METHODS:**

Eighty-two patients who underwent either ascending aortic replacement or aortic arch replacement between 2016 and 2022 in our institution were enrolled in the present study. Postoperative computed tomography scans were performed to detect fluid capsules with a diameter ≥3.0 cm and radiodensity ≤25 Hounsfield units. Patients who did and who did not develop PGS formation were compared. Variables with a statistically significant difference between these groups were included in a multiple logistic regression analysis along with other factors associated with PGS in the literature.

**RESULTS:**

The incidence rate of PGS was 14.6% (12/82). The average radiodensity of the mass was 16.6 ± 6.3 Hounsfield units. The average onset of PGS was 8.5 months post-surgery. Multivariate logistic regression analysis revealed that ejection fraction [odds ratio (OR): 1.25, 95% confidence interval (CI): 1.03–1.50, *P* = 0.021], aortic dissection (versus degenerative aortic aneurysm) (OR: 6.61, 95% CI: 1.35–32.4, *P* = 0.02) and warfarin use (OR: 6.67, 95% CI: 1.19–37.1, *P* = 0.03) significantly contributed to mediastinal PGS after thoracic aortic surgery.

**CONCLUSIONS:**

High ejection fraction, warfarin use and aortic dissection (versus degenerative aortic aneurysm) contributed significantly to mediastinal PGS formation after thoracic aortic surgery. Careful serial postoperative imaging studies and fluid analysis can be used to guide treatment plans.

**Clinical trial registration:**

UMIN-CTR (University hospital Medical Information Network-Clinical Trial Registry) Registration number: UMIN000050764.

**Figure:**
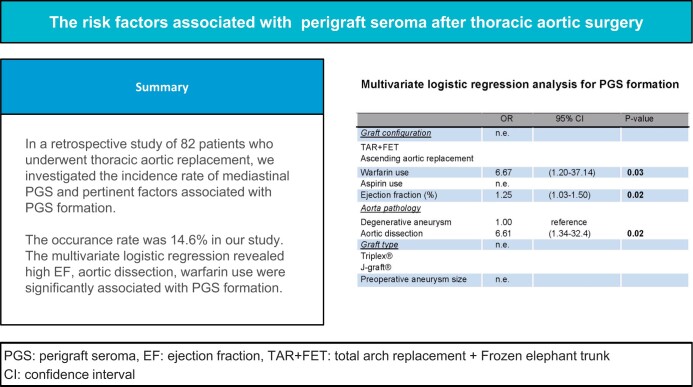

## INTRODUCTION

Perigraft seromas (PGSs) are associated with various vascular procedures, including peripheral bypass grafting, conventional and endovascular aortic repair. PGS is a persistent, sterile fluid accumulation close to an implanted graft that can appear following vascular surgeries. A fair number of PGS cases that developed after extra-anatomical bypass graft or abdominal aortic aneurysm repair have been reported in the past, and their underlying mechanisms and pertinent risk factors have been analysed. However, the mechanism and pertinent risk factors in mediastinal PGSs that develop after thoracic aortic surgery remain unclear. Here, we investigated the risk factors associated with mediastinal PGS. Additionally, this study provided a clinical review of mediastinal PGS after thoracic aortic surgery.

## PATIENTS AND METHODS

### Ethics statement

This study was approved by the Local Institutional Review Board (IRB) of Shinmatsudo Central General Hospital (Institutional Review Board registration number: 20220011 approved on 2/4/2023). Written informed consents were provided by all participants.

### Study population

Patients who underwent thoracic aortic surgery, either ascending aortic replacement or aortic arch replacement, with or without concomitant procedures, between 2016 and 2022 in our institution were enrolled in this study. Aortic pathology included aortic dissection as well as degenerative thoracic aneurysm. Emergency cases, cases using an elephant trunk and redo cases were included in this study. Patients complicated by deep sternal infection or PGS soon after initial surgery (within 2 months) were excluded from the study.

### Follow-up

Generally, patients were followed up in our clinic. A computed tomography (CT) scan was performed at 1, 3 and 6 months postoperatively and annually thereafter for evaluation. For patients followed up by another hospital, the corresponding physicians were contacted or patients or family were directly called by phone. Loss to follow-up was defined as patients who were lost to follow-up within 30 days following going home [1]. Accordingly, all patients were followed up.

### Definition of perigraft seroma

PGS is defined as a persistent, sterile fluid accumulation around a vascular prosthesis [2, 3]. The diagnosis of PGS needs an exclusion of other aetiology, such as infection, pseudoaneurysm and haematoma. Accordingly, CT scan criteria were required. The diagnosis was confirmed by utilizing a combination of radiodensity on the CT image, bacterial culture and laboratory and histological study (if possible), as follows:

- Perigraft fluid collection normally presents >3 months after surgery [4]; therefore, perigraft fluid formation immediately after surgery was not considered as seroma formation.
- A fluid capsule diameter of ≥3.0 cm and radiodensity on the CT scan ≤25 Hounsfield units (HU) [4].
- Negative inflammation markers in blood laboratory findings and blood cultures.
- No sign of a leak or pseudoaneurysm on contrast-enhanced CT scan.
- Absence of inflammatory changes, bubbles and wall enhancement in CT findings.
- A fatty preparation was injected through the nasogastric tube to rule out lymphorrhea in each suspected PGS case.

### Statistical analyses

To elucidate the pertinent factors associated with PGS formation, patients who did and those who did not develop PGS were compared. Statistically significant variables (*P* < 0.05) for PGS formation, based on the results of a comparison between the groups, as well as factors contributing to PGS formation based on the literature, were analysed via multivariate logistic regression analysis using stepwise inclusion methods. Fisher’s exact test was used to determine whether the 2 categorical variables were clearly related. The Mann–Whitney *U* test was used to compare the differences between 2 independent continuous variables while data were not normally distributed. Variables are presented as median and interquartile range. An unpaired *t*-test was used to determine whether there was a difference between the means of the continuous variables when the sample data were normally distributed. For normally distributed data, variables were expressed as the mean ± standard deviation. We used the Statistical Package for the Social Sciences Statistics (version 22.0; IBM Japan, Ltd, Tokyo, Japan) for statistical analyses.

## RESULTS

**Figure 1:**
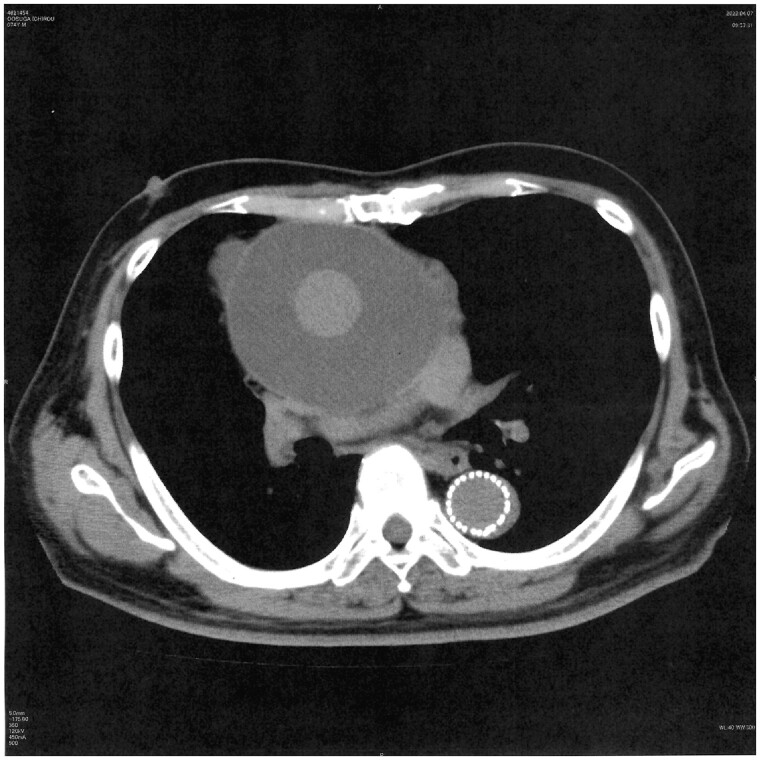
Enlarging perigraft seroma compressing the left pulmonary artery.

**Figure 2:**
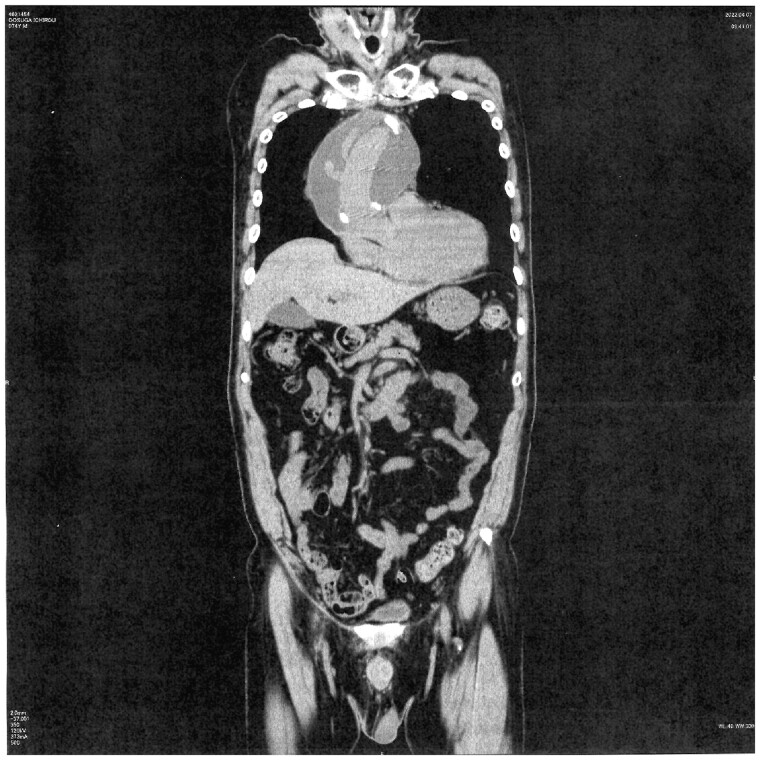
Perigraft seroma developed longitudinally from the bottom of the aortic root up to the aortic arch.

Approximately 14.6% (12/82) of patients developed PGS after either ascending aortic replacement or aortic arch replacement. The mean duration of follow-up was 40.5 ± 21.5 months (39.9 months [26.3–57.6 months]). The average radiodensity of the PGS mass was 16.6 ± 6.3 HU. The average onset of PGS was 8.5 months after surgery and was maintained thereafter. The average size of the PGS was 58.8 ± 14.7 mm. The masses tended to expand in size. As a result, the surrounding tissue, such as the left pulmonary artery, was compressed (Fig. 1). However, the prosthetic graft was not compressed. PGS developed along the longitudinal axis of the graft, from the aortic root to the aortic arch (Fig. 2). Surgical intervention, such as drainage or graft replacement, was not needed for most of the patients (10/12) during the observation period, although the PGS in those patients had also increased in size. Table 1 presents the baseline characteristics of the patients who developed PGS versus those without PGS. Patients with PGS formation showed a significantly higher ejection fraction (EF) than did those without PGS. Patients with aortic dissection were significantly more likely to develop PGS than were patients with degenerative aneurysms.

**Table 1:** Baseline characteristics: non-perigraft seroma patients versus perigraft seroma patients

	No PGS	PGS (+)	*P*-value
	Data (*N* = 70)	Data (*N* = 12)	
Male, *n* (%)	42 (60.0)	7 (58.3)	>0.999 a
Female, *n* (%)	28 (40.0)	5 (41.7)	
Age, *n* (%)	74.6 ± 8.9	71.4 ± 12.2	0.287 b
Body surface area (m^2^)	1.6 ± 0.2	1.6 ± 0.2	0.721 b
Hyperlipidaemia, *n* (%)	38 (55.1)	7 (58.3)	>0.999 a
Anticoagulation (W/D), *n* (%)	17 (24.3)	4 (33.3)	0.493 a
Warfarin, *n* (%)	10 (14.3)	4 (33.3)	0.205 a
Aspirin, *n* (%)	27 (37.5)	4 (40)	>0.999 a
Steroid, *n* (%)	3 (4.2)	1 (10)	0.412 a
Diuretics, *n* (%)	26 (36.1)	3 (30)	>0.999 a
Diabetes mellitus, *n* (%)	14 (20.0)	2 (16.7)	>0.999 a
Smoking, *n* (%)	24 (34.3)	6 (50.0)	0.341 a
Hypertension, *n* (%)	56 (80.0)	12 (100.0)	0.116 a
Peripheral vascular disease, *n* (%)	13 (18.6)	2 (16.7)	>0.999 a
Lung disease, *n* (%)	6 (8.6)	1 (8.3)	>0.999 a
Arrhythmia, *n* (%)	4 (5.7)	0 (0.0)	>0.999 a
Previous angina, MI, *n* (%)	8 (11.4)	1 (8.3)	>0.999 a
Cerebral vascular disease, *n* (%)	14 (20.0)	2 (16.7)	>0.999 a
Previous heart surgery, *n* (%)	9 (12.9)	1 (9.1)	>0.999 a
Preoperative albumin level, *n* (%)	4.1 ± 0.3	4.1 ± 0.4	0.888 b
Haemodialysis, *n* (%)	4 (5.7)	1 (8.3)	0.556 a
Serum creatinine (mg/dl)	0.9 [0.6, 1.2]	0.9 [0.8, 1.3]	0.455 c
Ejection fraction (%)	62.8 ± 7.1	67.4 ± 4.1	**0.032** b
Aortic valve, *n* (%)			0.716 a
Aortic stenosis (AS)	4 (5.7)	1 (8.3)	
Aortic regurgitation (AR)	14 (20.0)	3 (25.0)	
AS + AR/ASR	8 (11.4)	2 (16.7)	
Mitral valve, *n* (%)	7 (10.0)	3 (25.0)	0.159 a
Degenerative aneurysm, *n* (%)	42 (60.0)	3 (25.0)	**0.031** a
Aortic dissection (type A/B), *n* (%)	28 (40.0)	9 (75.0)	
Preoperative aneurysm size, *n* (%)	50.8 ± 8.0	50.9 ± 11.9	0.970 b

*P*-value: (a) unpaired *t*-test; (b) Fisher's exact test; and (c) Mann–Whitney *U* test. Values are presented as number (%) or mean (SD) or median [IQR].

ASR: aortic stenosis and regurgitation; IQR: interquartile range; anticoagulation (W/D): warfarin or direct oral anticoagulant; aortic dissection (type A/B): DeBakey type A or B; SD: standard deviation.

Bold represents the statistical significance.

**Figure 3:**
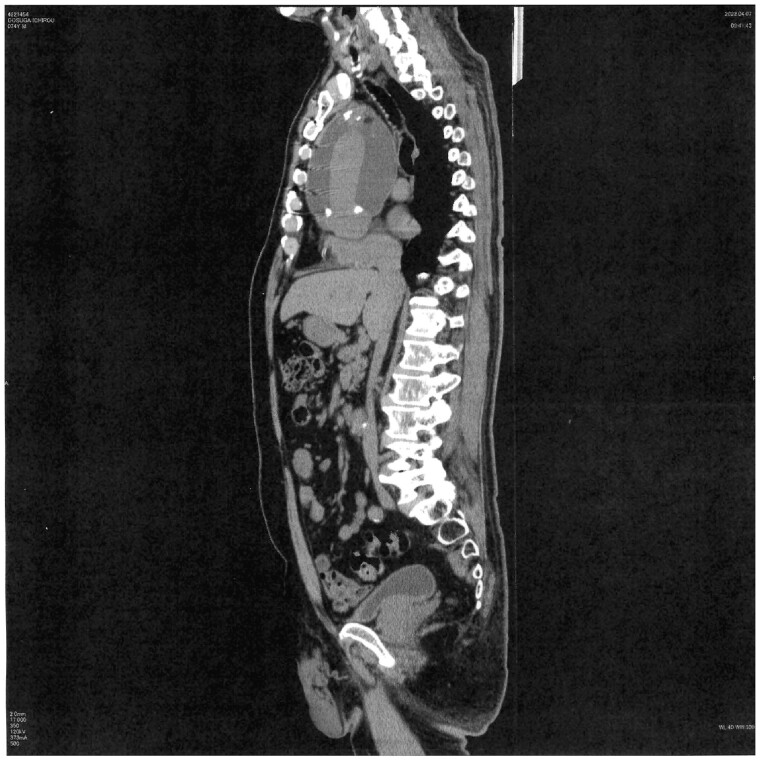
Large perigraft seroma attached to back of the sternum, creating a skin fistula.

**Figure 4:**
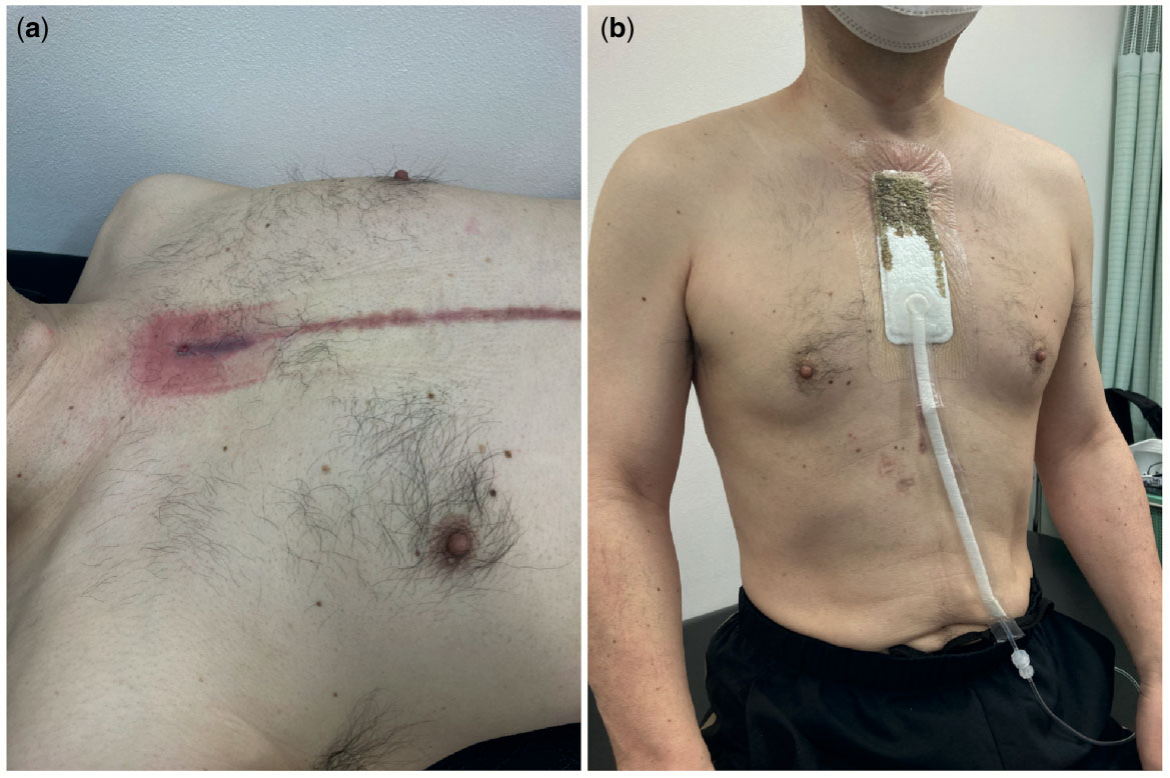
(**a**) Skin erosion due to fluid leakage through the perigraft seroma and skin fistula. (**b**) Wound-VAC system applied to the skin lesion to evacuate the fluid discharge.

Intraoperative findings and postoperative outcomes are presented in Table 2. Patients who developed PGS were dominated by the graft configuration with ascending aortic replacement (*P* = 0.054). The type of graft, Triplex (Terumo Corporation, Tokyo, Japan) versus J-graft (Japan Lifeline Co., Ltd, Tokyo, Japan), did not show a significant difference in distribution between patients with and without PGS formation; however, the Triplex graft was involved more in PGS formation more often than the J-graft (Triplex: 75% [9/12] versus J-graft: 25% [3/12]), although the difference was not significant. Multivariate logistic regression analysis revealed that EF [odds ratio (OR): 1.25, 95% confidence interval (CI): 1.03–1.50, *P* = 0.021], aortic dissection (versus degenerative aortic aneurysm) (OR: 6.61, 95% CI: 1.35–32.4, *P* = 0.02) and warfarin use (OR: 6.67, 95% CI: 1.19–37.1, *P* = 0.03) significantly contributed to mediastinal PGS after thoracic aortic surgery (Table 3). The use of aspirin, steroids and diuretics did not contribute to PGS formation in our study. Neither graft configuration nor graft type was significant for PGS formation in the logistic regression analysis (Table 3). The size of the mass in 2 PGS cases was increasing to the point where the round mass was attached to the back of the sternum (Fig. 3), forming a fistula between them. Occasionally, fluid was evacuated through a skin fistula (Fig. 4a), requiring vacuum-assisted closure of a wound, also known as wound-VAC placement (Fig. 4b). Out of 12 patients with PGS formation, wound-VAC was required for 2 cases (16.6% 2/12) as the PGS attached to the back of sternum was complicated by constant drainage.

**Table 2:** Intraoperative findings and postoperative outcomes

	PGS (−)	PGS (+)	
	*P* = 70	*P* = 12	
Graft type			>0.99 a
Triplex, *n* (%)	54 (77.1)	9 (75.0)	
J-graft, *n* (%)	16 (22.9)	3 (25.0)	
Graft configuration			0.054 a
TAR + FET, *n* (%)	45 (64.3)	4 (33.3)	
AAR, *n* (%)	21 (30.0)	8 (66.7)	
Others, *n* (%)	4 (5.7)	0 (0.0)	
Operation time (min)	270.0 [244.0, 360.0]	260.0 [248.3, 350.0]	0.773 c
OSG length (mm)	60.0 [60.0, 90.0]	60.0 [60.0, 82.5]	0.446 c
Haemostatic agent (%)	28 (40.0)	9 (75.0)	0.031 a
Concomitant op, *n* (%)	16 (22.8)	2 (16.7)	0.722 a
Arrest time (min)	167.0 [148.0, 206.0]	162.0 [150.8, 192.3]	0.973 c
CPB time (min)	201.0 [173.3, 247.3]	191.5 [175.3, 203.5]	0.609 c
Intubation length (days)	2.7 [1.7, 5.3]	2.4 [1.7, 3.7]	0.61 c
ICU stay (day)	7.0 [5.0, 13.3]	6.0 [4.0, 9.8]	0.42 c
Complications, *n* (%)	34 (48.6)	4 (33.3)	0.367 a
Stroke	13 (18.1)	0 (0)	0.351 a
Pneumonia	4 (5.6)	0 (0)	>0.999 a
Renal failure	2 (2.8)	0 (0)	>0.999 a
Urinary tract infection	5 (6.9)	1 (10)	0.554 a
Thirty-day mortality			
Death, *n* (%)	3 (4.3)	0 (0.0)	>0.99 a
Five-year mortality			
Death, *n* (%)	10 (21.3)	2 (50.0)	0.232 a

*P*-value: (a) unpaired *t*-test; (b) Fisher's exact test; and (c) Mann–Whitney *U* test. Values are presented as number (%) or mean (SD) or median [IQR].

AAR: ascending aortic replacement; CPB: cardiopulmonary bypass; ICU: intensive care unit; IQR: interquartile range; op: operation; OSG: open stent graft; PGS: perigraft seroma; SD: standard deviation; TAR + FET: total arch replacement + frozen elephant trunk.

**Table 3:** Multivariate logistic regression analysis for mediastinal perigraft seroma

	OR	95% CI	*P*-value
Graft configuration	n.e.		
TAR + FET			
Ascending aortic replacement			
Warfarin	6.67	1.20–37.14	**0.03**
Aspirin	n.e.		
Ejection fraction (%)	1.25	1.03–1.50	**0.02**
Aorta pathology			
Degenerative aneurysm	1.00	Ref	
Aortic dissection	6.61	1.34–32.4	**0.02**
Graft type	n.e.		
Triplex			
J-graft			
Preoperative aneurysm size	n.e.		

CI: confidence interval; n.e.: not entered; OR: odds ratio; TAR + FET: total arch replacement + frozen elephant trunk.

Bold represents the statistical significance.

## DISCUSSION

There has been no previous original study to analyse the risk factors associated with mediastinal PGS after thoracic aortic surgery. Using multivariate logistic regression analysis, we demonstrated that high EF, aortic dissection (versus degenerative aortic aneurysm) and warfarin use contributed significantly to mediastinal PGS formation after thoracic aortic surgery. In our study, the incidence of mediastinal PGS formation after thoracic aortic surgery was 14.6%. The incidence of PGS in a previously reported series of abdominal aortic aneurysms was up to 18% [4]. Spartera *et al.* [5] reported that 19% of patients who had undergone thoracic aortic surgery had >3.5 cm of perigraft fluid collected by 1-month postoperatively. Mediastinal PGS formation has been presumed to be rare, and no systematic review on this topic, particularly for mediastinal PGS following thoracic aortic surgery, has been reported, suggesting the novelty of the present study. The asymptomatic nature and deep anatomical location of PGS preclude easy detection by physical examination, which may be the reason for their low incidence. Seromas are relatively common in superficially placed grafts (such as axillofemoral and femorofemoral bypasses), whereas deep graft seromas occur much less frequently, and seromas have been described mainly in abdominal aorta grafts [4, 6]. In general, PGS after abdominal aortic aneurysm repair often causes some symptoms due to the formation of space-occupying lesions; however, mediastinal PGS after thoracic aortic aneurysm repair is not always accompanied by symptoms due to the larger free space in the mediastinum.

In general, the diagnosis of PGS is made by excluding other differential diagnoses such as haematoma, infection and lymphorrhea [6]. Kadakol *et al.* defined PGS as perigraft fluid collection that presents at >3 months after surgery, with a diameter of >3.0 cm and radiodensity on CT scans <25 HU. Therefore, patients with perigraft fluid formation immediately after surgery were excluded from our study [4]. In our cohort, the average period from surgery to PGS onset was 8.5 months and the average radiodensity of mass on CT was 16.6 ± 6.3 HU. Our cases met all 3 above criteria. In graft infection and lymphorrhea, radiodensity is >20 HU; therefore, CT ruled out haemorrhage/haematoma (50 HU) but not infection and lymphorrhea completely. It is relatively difficult to rule out lymphorrhea as it compresses the surrounding tissue, which is similar to seroma and lacks other specific symptoms.

Generally, the accumulation of low-density perigraft fluid is often due to infection and needs to be excluded. Bleeding due to anastomotic dehiscence without infection is rare but needs to be excluded as well. Infection and haematoma are frequently occurred in the early postoperative phase [6]. In about 50% of cases, the cause of low-density perigraft fluid accumulation has not been identified [7], and patients remain asymptomatic. It is hypothesized that late-onset perigraft low-attenuation fluid collections result from a postoperative seroma or inflammatory oedema that developed due to an allergic reaction to the graft material [2, 8]. Our data showed that the average period from surgery to PGS onset was 8.5 months, which supports the aforementioned theory.

Kadakol *et al.* [4] reported that multivariate analysis identified diabetes, smoking, anticoagulation and bifurcated graft reconstruction for abdominal aortic aneurysm repair as preoperative risk factors for PGS formation. Using multivariate logistic regression analysis, we demonstrated that high EF, aortic dissection (versus degenerative aortic aneurysm) and warfarin use contributed significantly to mediastinal PGS formation after thoracic aortic surgery. In general, aortic dissection involves more inflammation at the aortic wall than does a degenerative aortic aneurysm; this may trigger the PGS formation process. Local haemostatic agents such as Bioglue were utilized in all aortic dissection cases, which may also have contributed to PGS formation. Aortic wall inflammation and fibrin leakage from the polytetrafluoroethylene (PTFE) graft have been reported to cause PGS [9]. Warfarin use has been a known risk factor for PGS formation [4]. Among several pertinent factors, anticoagulation was strongly related to PGS formation. In addition, the action of anticoagulant therapy is thought to be related to ultrafiltration, as it can inhibit microthrombus formation in the graft porosity [4]. After discontinuation of anticoagulants, the PGS was reported to decrease in size [4]. Two cases of diminishing size of PGS by discontinuation of antiplatelet therapy following surgical repair of abdominal aortic aneurysms [10] have been reported, although our data failed to show a significant correlation between antiplatelet treatment and PGS. Bolton and Cannon [11] conducted animal research investigating the haemodynamic status to determine the aetiology of PGS formation using PTFE grafts. High blood-flow status and acute bending grafts were determined to be the factors related to PGS formation. High blood-flow status in the setting of an acute bending graft can lead to a haemodynamic status with a high concentration of blood corpuscle components in the central flow and a low concentration in the peripheral flow; thus, blood viscosity in the peripheral flow would be reduced to the point where serum could leak through the graft. We showed that a high EF contributed significantly to PGS formation. This may also be linked to a high blood-flow status.

The biological mechanism underlying PGS remains uncertain. However, several mechanisms have been reported, including an immunological response (allergy) [8], low-grade infection (bacterial biofilm) [2] and discharge of fluids through the graft (ultrafiltration) [12], as the graft surface acts as an ultrafiltration membrane. Ultrafiltration is induced by the suppression of fibroblast growth, which prevents connective tissue from adhering to the graft. Blumenberg *et al.* [2] showed the microscopic findings of incomplete attachment of the PTFE graft into the surrounding tissue. A scant connective tissue in the surrounding graft was observed in patients with PGS. Sladen *et al.* [13] showed that serum from patients with PGS prevented fibroblast from proliferating *in vitro*. In general, the growth of endothelial cells towards the graft and incorporation of the graft into the surrounding tissue prevent serum leakage. If this process failed or was delayed, plasma components would leak through the graft pore [14]. Second, immunological responses, such as allergy to graft material, may trigger seroma formation [8, 12]. Third, a bacterial biofilm is a silent infection with the absence of systemic sepsis and can present as a fluid-filled cavity surrounding the graft. It is insidious and may mimic the sterile process [12].

Whether the graft material is responsible for PGS formation remains controversial [15]. We failed to determine whether graft configuration (ascending aortic replacement or total arch replacement) or graft type (Triplex or J-graft) contributed to PGS formation. Blumenberg *et al.* [2] reported that knitted Dacron grafts were most frequently used in PGS cases (55%), followed by PTFE grafts (34%) and woven Dacron (7%). Others have reported that all PGS cases are related to the PTFE grafts [4, 12]. Kadakol *et al.* found that PTFE grafts are more frequently involved in PGS formation than are Dacron grafts [4]. We demonstrated that the Triplex graft was involved more often than the J-graft in PGS formation (Triplex: 75% [9/12] versus J-graft: 25% [3/12]), although the difference was not statistically significant. Triplex has a three-layered structure with a non-porous middle layer commonly referred to as elastomer. It does not require pre-clotting and has shown to suppress immunological reactions. The 3 layers enable haemostasis around the anastomosis [16]. Theoretically, it poses no risk of PGS development due to ultrafiltration. The aetiology of PGS due to the Triplex graft remains unknown. One case report of mediastinal PGS after total arch replacement using the Triplex graft has been published [17]. However, no clinical reviews of a considerable number of PGS cases after thoracic aortic surgery using the Triplex graft are available. In addition, graft handling during surgery can impact graft integrity, leading to fluid seeping through the graft wall. Examples include wetting the graft with organic solvents [2], pressurizing the graft with irrigation solutions [18] and soaking it in an antibiotic solution [19].

Various treatments are used for seroma such as serial aspiration, incision and drainage and graft replacement. However, the results are sometimes uncertain [20]. The treatment aims to alleviate symptoms, reduce the chance of recurrence and avoid complications, such as skin erosion, secondary graft infection and rupture [2, 3, 21]. Sac enlargement due to PGS after open surgery is considered to be a benign condition, and most physicians suggest an observational strategy as long as patients remain asymptomatic. Surgical intervention is reserved for symptomatic cases [22]. Accordingly, we initially used an observation strategy, as mediastinal PGS after thoracic aortic repair was reported to be rare, and symptomatic PGS requiring surgical intervention was considered extremely rare. A few case studies have shown that large mediastinal PGS may cause compressive symptoms, potentially leading to airway obstruction or tamponade pathophysiology in the heart, which may require surgical intervention [18, 23, 24]. Some of our patients experienced pulmonary artery compression caused by PGS. Fortunately, surgical intervention has not been required thus far in the observation period in our cohort. Two cases developed constant fluid leakage through PGS that adhered to the sternum, requiring wound-VAC placement. We observed shrinkage of the PGS over time. Treating large PGS-forming fistulas between the skin and mediastinum through a median sternotomy may be effective. Fluid aspiration may be effective for the diagnosis and treatment of PGS, although we did not perform the puncture procedure, due to anatomical difficulty. Distinguishing whether fluid collection is transudate or exudate by needle aspiration may be helpful for determining a management plan. If fluid analysis reveals transudate fluid, the mechanism of PGS would be ultrafiltration, whereas if it showed exudate fluid, the cause of PGS would be biofilm, requiring graft replacement [12]. However, needle aspiration can provide only temporary relief for patients, as PGS re-emerges soon after aspiration if the membrane tissue is not removed [22]. The treatment option of replacing the original graft with a new one made of different material has been reported to be effective [10, 25]. Recent advancements in surgical intervention include fibrin glue applied to the graft [6], wrapping the graft using omentum [26] and endovascular relining of the prosthesis [27–29].

Although PGS is rare, vascular surgeons dealing with the graft replacement may encounter this adverse event once in an entire career. For them to provide the best treatment, fundamental knowledge regarding PGS is necessary.

### Limitations

This was a retrospective single-arm study in a small number of patients. However, logistic regression analysis revealed significantly associated factors, which are worth reporting. Large number of patients with long-term follow-up is required to confirm our findings. Moreover, we did not perform any histological examination for a definitive diagnosis of PGS. Missed diagnoses of lymphatic perigraft fluid collection for PGS may have occurred. However, a fatty preparation was given through the nasogastric tube for each suspected PGS case and demonstrated no chyle leakage.

## CONCLUSION

We demonstrated that high EF, aortic dissection (versus degenerative aortic aneurysm) and warfarin use significantly contributed to mediastinal PGS after thoracic aortic surgery. A fair proportion of PGS was related to use of the Triplex graft in our cases; however, the aetiology was unknown. The first strategy for mediastinal PGS after thoracic aortic surgery is conservative observation, unless symptomatic PGS necessitates surgical interventions. Careful monitoring by using serial imaging studies and fluid analysis is paramount.

**Conflict of interest:** none declared.

## Data Availability

The data analysed in this article may be shared upon request with the corresponding author.

## ABBREVIATIONS

CI: Confidence interval
CT: Computed tomography
EF: Ejection fraction
HU: Hounsfield units
OR: Odds ratio
PGS: Perigraft seroma
PTFE: Polytetrafluoroethylene
